# SNOSite: Exploiting Maximal Dependence Decomposition to Identify Cysteine S-Nitrosylation with Substrate Site Specificity

**DOI:** 10.1371/journal.pone.0021849

**Published:** 2011-07-15

**Authors:** Tzong-Yi Lee, Yi-Ju Chen, Tsung-Cheng Lu, Hsien-Da Huang, Yu-Ju Chen

**Affiliations:** 1 Department of Computer Science and Engineering, Yuan Ze University, Chung-Li, Taiwan; 2 Institute of Chemistry, Academia Sinica, Taipei, Taiwan; 3 Institute of Bioinformatics and Systems Biology, National Chiao Tung University, Hsin-Chu, Taiwan; 4 Department of Biological Science and Technology, Hsin-Chu, Taiwan; University of South Florida, United States of America

## Abstract

S-nitrosylation, the covalent attachment of a nitric oxide to (NO) the sulfur atom of cysteine, is a selective and reversible protein post-translational modification (PTM) that regulates protein activity, localization, and stability. Despite its implication in the regulation of protein functions and cell signaling, the substrate specificity of cysteine S-nitrosylation remains unknown. Based on a total of 586 experimentally identified S-nitrosylation sites from SNAP/L-cysteine-stimulated mouse endothelial cells, this work presents an informatics investigation on S-nitrosylation sites including structural factors such as the flanking amino acids composition, the accessible surface area (ASA) and physicochemical properties, i.e. positive charge and side chain interaction parameter. Due to the difficulty to obtain the conserved motifs by conventional motif analysis, maximal dependence decomposition (MDD) has been applied to obtain statistically significant conserved motifs. Support vector machine (SVM) is applied to generate predictive model for each MDD-clustered motif. According to five-fold cross-validation, the MDD-clustered SVMs could achieve an accuracy of 0.902, and provides a promising performance in an independent test set. The effectiveness of the model was demonstrated on the correct identification of previously reported S-nitrosylation sites of *Bos taurus* dimethylarginine dimethylaminohydrolase 1 (DDAH1) and human hemoglobin subunit beta (HBB). Finally, the MDD-clustered model was adopted to construct an effective web-based tool, named SNOSite (http://csb.cse.yzu.edu.tw/SNOSite/), for identifying S-nitrosylation sites on the uncharacterized protein sequences.

## Introduction

S-nitrosylation is a reversible post-translational modification (PTM) by covalent modification on the thiol group of cysteine (Cys) residues by nitric oxide (NO). Emerging evidences suggest that S-nitrosylation plays an important role in NO-related and redox pathway, especially in immune, cardiovascular, neuronal, and plant systems [1], [2], [3], [4], [5], [6]. Moreover, different S-nitrosylation level and targets modulate the protein activity, localization, and stability [7], [8], [9] and further regulate the pathophysiological events, such neurodegenerative diseases and cancers [10], [11], [12]. Due to the labile nature and low abundance of S-nitrosylation *in vivo*, the detail characteristics and mechanisms of S-nitrosylation still remain to be clarified. To our knowledge, the protein database of human, mouse, or rat possess only approximate 2% cysteine residues, however, not all cysteine residues on proteins can be S-nitrosylated by NO. Accumulating studies reveal that the cysteine residue, having low pKa or exposed thiol group on protein surface, is more accessible by NO modification [8], [13].

With the increasing number of experimentally verified S-nitrosylation sites by proteomics advancement, several studies have revealed the S-nitrosylated cysteine residues may locate on acid-base motif, flanking with acidic (Aspartate or Glutamate) and basic (Arginine, Lysine, or Histidine) amino acids, or embed into the hydrophobic area [14], [15], [16], [17], [18], [19]. Based on the structural analysis of S-nitrosylation on proteins, Marino *et al.* have revealed a modified acid-base motif, which is located more distantly to the cysteine and has its charged groups exposed [20]. However, whether other potential novel consensus S-nitrosylation motifs are present on proteins is not clear. The critical determinant of other structural component needs to be analyzed. Due to the labile nature of the S-NO bond and the low abundance of endogenously *S*-nitrosylated proteins *in vivo*, however, the unambiguous identification of *S*-nitrosylated proteins and *S*-nitrosylation sites remains challenging by commonly used proteomic technology [6], [14], [15], [21], [22], [23], [24]. From the structural point of view, thus, it is important to develop a method for the efficient and site-specific detection of protein *S*-nitrosylation, experimentally or computationally.

To date, approximately one thousand of proteins have been identified to related to S-nitrosylation in different biological systems [25], yet the specificity of S-nitrosylation sites are not completely understood. Using *in silico* prediction, GPS-SNO, has been proposed to computationally identify S-nitrosylation sites, with a sensitivity of 53.57% and a specificity of 80.14% [26]. Recently, we have developed an S-alkylating biotin switch method and identified 586 S-nitrosylation sites corresponding to 384 S-nitrosylated proteins in SNAP/L-cysteine-stimulated mouse endothelial cells [19]. Using motif-X algorithm, 7 of 10 potential consensus motifs having local hydrophobicity at +2 position, containing acid-basic amino acids flanking with the central S-nitrosylating cysteine residues, were artificially extracted from ∼30% S-nitrosylated peptides [19], [27]. Considering that the majority of the S-notrisylaiton sites did not match to the motif, other unknown structural factors must be taken into consideration. To further investigate potential S-nitrosylation motifs in primary amino acid sequence, the *in silico* characterization, i.e. amino acid composition, accessible surface area (ASA), and physicochemical properties, of protein S-nitrosylation sites is needed for distinguishing the S-nitrosylation sites from non-S-nitrosylation sites.

This work investigates site-specific characteristics for 586 experimentally verified S-nitrosylation sites [19] and applies maximal dependence decomposition (MDD) [28] to identify the potential substrate motifs of S-nitrosylation. With the application of MDD, a large group of aligned sequences can be moderated into subgroups that capture the most significant dependencies between positions. Support vector machine (SVM) is applied to generate the predictive model for each MDD-clustered subgroup. By further evaluation using five-fold cross-validation, the SVM models trained with MDD-clustered subgroups could improve predictive accuracy when compare to the model without the application of MDD clustering. Moreover, the experimental S-nitrosylation data from GPS-SNO (independent set) are used to test the effectiveness of the models that achieve the best accuracy in cross-validation. Finally, the models with MDD clustering method are adopted to implement an effective web-based tool, named SNOSite, for identifying cysteine S-nitrosylation sites. Two experimentally verified S-nitrosylated proteins, which were not included in training set, demonstrate the effectiveness of SNOSite. The *in silico* identification has potential for characterizing S-nitrosylation sites before experiments are performed.

## Materials and Methods

### Data preprocessing of training set and independent test set

With the high-throughput S-alkylating biotin switch method, a total of 586 S-nitrosylation sites corresponding to 384 S-nitrosylated proteins were experimentally identified in SNAP/L-cysteine-stimulated mouse endothelial cells for 30 minutes [19]. The experimental data on S-nitrosylated cysteines constituted the positive data of training set, and non-S-nitrosylated cysteines in the experimentally validated S-nitrosylated proteins constitute the negative data of training set, respectively. As shown in Table 1, 586 positive data and 2728 negative data in 384 S-nitrosylated proteins were obtained. This study focused on the sequence-based analysis of substrate specificity of cysteine S-nitrosylation. Subsequently, the identified motifs would be evaluated the ability to distinguish the S-nitrosylated cysteine from the non-S-nitrosylated cysteine, based on cross-validation.

**Table 1:** The statistics of experimentally verified S-nitrosylation sites in training set and independent test set.

Data set	Species	Number of S-nitrosylated proteins	Number of S-nitrosylated cysteine	Number of non-S-nitrosylated cysteine
**Training set**(Chen *et al.*)	Mouse	384	586	2,728
**Independent test set**(GPS-SNO)	Multiple	327	479	2,501

As for classification, the prediction performance of the trained models may be overestimated owing to the over-fitting of a training set. The experimental S-nitrosylation sites that collected from GPS-SNO were regarded as the independent test set, which consist of 504 S-nitrosylated cysteines (positive data) in 327 experimental S-nitrosylated proteins. Similar to the extraction of a negative data of training set, a total of 2581 non-S-nitrosylated cysteines were regarded as a negative data of independent test set. After the cross-validation of training set, the independent test set was evaluated by using the trained model with the highest accuracy. However, the positive data of independent test set may include the sequences that are homologous to training data. To prevent any overestimation of predictive performance, the homologous sequences between training set and independent test set were removed. With reference to the reduction of the homology of the training set in MASA [29], two S-nitrosylated protein sequences with more than 30% identity were defined as homologous sequences. Then, two homologous sequences were specified to re-align the fragment sequences using a window length of 2*n*+1, centered on the S-nitrosylation sites using BL2SEQ [30]. For two fragment sequences with 100% identity, only one S-nitrosylation site on homologue fragment sequence in training set was kept while the other in test set was discarded. The non-homologous negative data were generated using the same approach as positive one. After the homology reduction, the non-homologous independent test set contained 479 positive sites and 2501 negative sites.

### Features investigation

Besides the composition of flanking amino acids (AA), the accessible surface area (ASA) and physicochemical properties around the S-nitrosylation sites were also investigated. Amino acids sequences with a S-nitrosylation site or cysteine in the center were extracted from positive and negative training sets, respectively, using a window of length 2*n*+1 varying from four to ten. Different values of *n* were used to determine the optimal window length. With reference the method of SulfoSite [31], the positional weighted matrix (PWM) of amino acids around the S-nitrosylated cysteines was determined using non-homologous training data. The positional weighted matrix (PWM) specified the relative frequency of amino acids that surround the S-nitrosylation sites, and was utilized in encoding the fragment sequences. A matrix of *m*×*w* elements was used to represent each residue of a training dataset, where *w* stands for the window size and *m* consists of 21 elements including 20 types of amino acids and one for terminal signal. In addition, WebLogo [32], [33] is adopted to generate the graphical sequence logo for the relative frequency of the corresponding amino acid at each position around the S-nitrosylation sites.

A side-chain of amino acid that undergoes post-translational modification prefers to be accessible on the surface of a protein [34]. Thus, the solvent-accessible surface area (ASA) was considered to evaluate the characteristics of S-nitrosylation sites. Since most of the experimental S-nitrosylated proteins do not have corresponding protein tertiary structures in PDB [35], an effective tool, RVP-Net [36], [37], is applied to compute the ASA value from the protein sequence. RVP-net applied a neural network to predict the real ASA of residues based on information about their neighborhood, with a mean absolute error of 18.0–19.5%, defined as the absolute difference between the predicted and experimental values of relative ASA per residue [37]. The computed ASA is the percentage of the solvent-accessible area of each amino acid on the protein. The full-length protein sequences with experimentally identified S-nitrosylation sites are inputted to RVP-Net to compute the ASA value of all of the residues. The ASA values of amino acids around the S-nitrosylation sites are extracted and normalized to be between zero and one.

A previous work has utilized 31 informative physicochemical properties to identify protein ubiquitylation sites [38]. To investigate the characteristics of S-nitrosylation sites in comprehensive deal, 531 physicochemical properties, that were extracted from version 9.1 of AAindex [39], are evaluated the ability to distinguish the S-nitrosylation sites from the non-S-nitrosylation sites. AAindex [39] includes many published indices that specify the physicochemical properties of amino acids. Since each physicochemical property of the amino acids is specified by a set of 20 numerical values, the amino acids around the S-nitrosylation sites can be encoded according to the values associated with each physicochemical property. In order to identify the significant physicochemical properties, a measurement of F-score [40] has been applied to calculate a statistical value for each position surrounding S-nitrosylation sites. The F-score of the *i*th physicochemical feature is defined as:
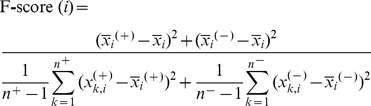
(1)where 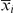, 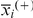 and 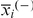 denote the average value of the *i*th feature in whole, positive, and negative data sets, respectively; 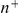 denotes the number of positive data set and 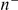 denotes the number of negative data set; 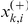 denotes the *i*th feature of the *k*th positive instance, and 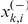 denotes the *i*th feature of the *k*th negative instance [40].

### Data clustering by maximal dependence decomposition

**Figure 1:**
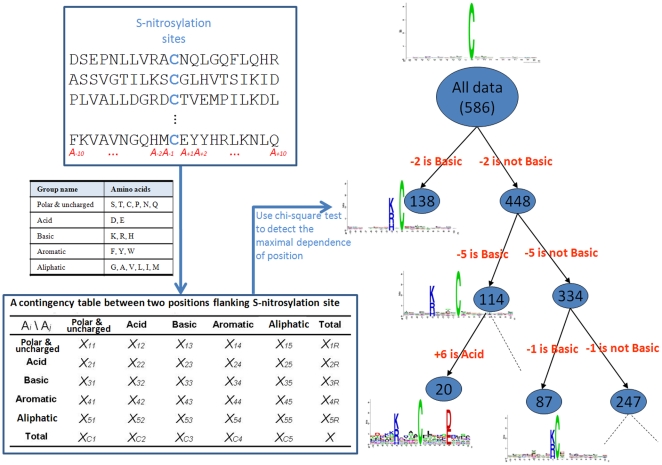
The flowchart of MDD clustering.

The aim of this study is to investigate the motifs of S-nitrosylation sites based on the amino acid sequences. Due to the difficulty of detecting the conserved motifs for the sequence data with a larger size, this work applies maximal dependence decomposition (MDD) [28] to cluster all sequences of S-nitrosylation site into subgroups, which have obvious motifs. MDD is a methodology to group a set of aligned signal sequences to moderate a large group into subgroups that capture the most significant dependencies between positions [41]. In previous study [28], MDD is firstly proposed to group the splice sites during the identification process of splice site prediction. However, in this work, we group protein sequences instead of nucleotides. MDD adopts chi-square test 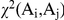 to evaluate the dependence of amino acid occurrence between two positions *A_i_* and *A_j_* that surround the S-nitrosylated cysteines. In order to extract the motifs that have conserved biochemical property of amino acids when doing MDD, we categorize the twenty types of amino acids into five groups such as aliphatic, polar and uncharged, acid, basic, and aromatic groups, as the grouping given in Table S1 (Supplementary Materials). Then, a contingency table of the amino acids occurrence between two positions is constructed, as presented in Fig. 1. The chi-square test is defined as:
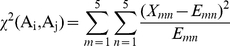
(2)where *X_mn_* represents the number of sequences that have the amino acids of group *m* in position *A_i_* and have the amino acids of group *n* in position *A_j_*, for each pair (*A_i_*, *A_j_*) with *i*≠*j*. *E_mn_* is calculated as 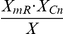, where *X_mR_* = *X_m1_*+…+*X_m5_*, *X_Cn_* = *X_1n_*+…+*X_5n_*, and *X* denotes the total number of sequences. If a strong dependence are detected (defined as a *X^2^* value is larger than 34.3, corresponding to a cutoff level of *P* = 0.005 with 16 degrees of freedom) between two positions, then proceed as described by Burge and Karlin [28]. After the detection of maximal dependence of flanking positions, as the example illustrated in Fig. 1, position −2 has the maximal dependence with the occurrence of basic amino acids. Subsequently, all data can be divided into two subgroups: one has the occurrence of basic amino acids in position −2 and the other does not have the occurrence of basic amino acids in position −2. The MDD clustering is a recursively process to divide the positive sets into tree-like subgroups. When applying MDD to cluster the sequences of a positive set, a parameter, i.e., the maximum-cluster-size, should be set. If the size of a subgroup is less the maximum-cluster-size, the subgroup will not be divided any more. The MDD process terminates until all the subgroup sizes are less than the value of maximum-cluster-size.

### Model learning and evaluation

The support vector machine (SVM) is applied to generate computational models that incorporate the encoded amino acids, accessible surface area and physicochemical properties. Based on binary classification, the concept of SVM is to map the input samples into a higher dimensional space using a kernel function, and then to find a hyper-plane that discriminates between the two classes with maximal margin and minimal error. A public SVM library, LibSVM [42], is used to train the predictive model with positive and negative training sets, which are encoded with reference to various training features. The radial basis function (RBF) 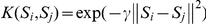 is selected as the kernel function of SVM. Cross-validation is important to the application of the predictor [43]. Predictive performance of the constructed models is evaluated by performing *k*-fold cross validation. The training data is divided into *k* groups by splitting each dataset into *k* approximately equal sized subgroups. During cross-validation, each subgroup is regarded as the validation set in turn, and the remainder is regarded as the training set. Next, the following measures of predictive performance of the trained models are defined. Precision (Pr) = TP/(TP+FP), Sensitivity (Sn) = TP/(TP+FN), Specificity (Sp) = TN/(TN+FP), Accuracy (Acc) = (TP+TN)/(TP+FP+TN+FN), Balanced Accuracy (BAcc) = (Sn+Sp)/2, and Matthews Correlation Coefficient (MCC) = 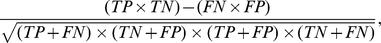 where TP, TN, FP and FN represent the numbers of true positives, true negatives, false positives and false negatives, respectively. Additionally, the parameters of the predictive models, window length, cost, and gamma value of the SVM models are optimized to maximize predictive accuracy. Finally, the window size and features that yield the highest accuracy are employed to construct predictive models for independent test.

## Results

### Positively charged and higher solvent accessible amino acids neighboring with the S-nitrosylated cysteines

**Figure 2:**
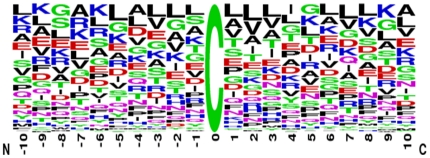
Frequency plot of sequence logo of S-nitrosylation sites with 21-mer window length.

**Figure 3:**
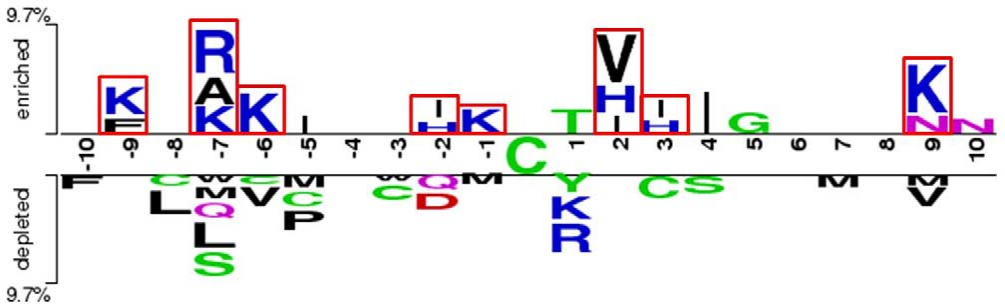
The compositional biases of amino acids around S-nitrosylation sites compared to the non-S-nitrosylation sites. The amino acids that are significantly enriched or depleted (*P*-value<0.05) around S-nitrosylation sites are presented.

This study focuses on the sequence-based analysis of substrate specificity for S-nitrosylation. To preliminarily evaluate the amino acid frequency neighboring the S-nitrosylated cysteine, the non-homologous S-nitrosylated cysteine is centered on position 0, and the flanking amino acids (−10∼+10) are graphically visualized as sequence logos. With the frequency plot of sequence logo representation given in Figure 2, no significant amino acids having high frequency is surrounding to the S-nitrosylation sites. In order to further explore the difference of amino acid composition between positive data and negative data, we applied a web-based tool TwoSampleLogo [44], that detects and displays statistically significant differences in position-specific symbol compositions between two sets of multiple sequence alignments. Figure 3 presents the position-specific difference of amino acid compositions between S-nitrosylation sites (586 sequences) and non-S-nitrosylation sites (2728 sequences). It reveals that the most pronounced feature of S-nitrosylation sites is the abundance of charged amino acids, especially the positively charged Lysine (K), Arginine (R), and Histidine (H), at positions −9, −7, −6, −2, −1, +2, +3, and +9. Another featured characteristic is the depletion of neural amino acids, such as L, V, P, M, C, and S, locating centrally around position −7. The results revealed that the distant amino acids in sequence, which may be close to S-nitrosylation cysteines in three-dimensional structure, have notable difference between S-nitrosylation sites and non-S-nitrosylation sites. Another interesting feature is the absence of positively charged residues at position +1 that is immediately adjacent to the S-nitrosylation sites. For instance, as shown in the lower pane of Figure 3, the K and R are depleted at position +1. In comparison with the 21 motifs (Table S2 in Supplementary Materials) detected by motif-X [27], the positively charged amino acids were also absent at position +1 in previous study. Moreover, the positively charged K at position −9, −6, −5, +9 and R and H at position −7, −6, and +2 were also present (Table S2). The result not only consisted with the sequence analysis by motif-X but also indicated that the positive charged amino acids surrounding the S-nitrosylated cysteines may play an important role for S-nitrosylation.

**Figure 4:**
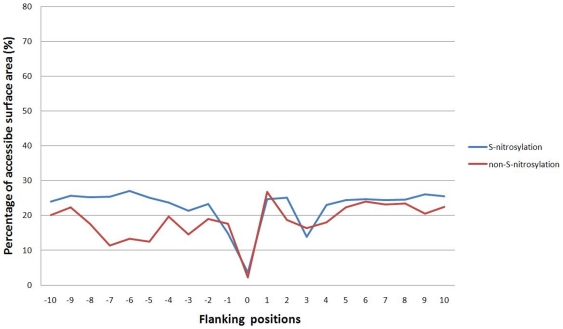
Comparison of average percentage of ASA in the 21-mer window (−10∼+10) between S-nitrosylation and non-S-nitrosylation sites.

Besides composition of amino acids, we further analyze the correlation of solvent accessible surface area (ASA) and S-nitrosylation sites. Because most of the experimentally verified S-nitrosylated proteins do not have corresponding protein tertiary structures in Protein Data Bank (PDB) [35], RVP-Net [36], [37], an ASA prediction tool that has been demonstrated to provide accurate ASA values similar to those observed in the protein tertiary structure [29], was applied to compute the ASA value of each residue in the protein sequence. Figure 4 presented the comparison of average percentage of ASA in the 21-mer window (−10∼+10) between S-nitrosylation and non-S-nitrosylation sites. This analysis showed that the cysteine residues have the lowest ASA on both S-nitrosylated or non-S-nitrosylated cysteines, suggesting low preference of solvent accessibility in S-nitrosylation sites. Moreover, the adjacent amino acids neighboring the centered S-nitrosylation sites have relatively higher preference of solvent-accessible surface area than that of non-S-nitrosylation sites, especially in the region of upstream sequences (−10∼−2). In particular, the positions −5, −6 and −7 have more obvious difference, which are also the locations for positive amino acids shown in Figure 3. Interestingly, the average percentage of ASA is particularly low at positions (−1 and +3) that are adjacent to the S-nitrosylation sites, suggesting, again, that the adjacent amino acids may regulate the S-nitrosylation on cysteine residues due to relative surface solvent accessibility.

### Cross-validation of characteristics for flanking amino acids and S-nitrosylation sites

To determine what window lengths and features can perform best to identify the S-nitrosylation sites, the predictive models are trained with various window lengths and various features and are evaluated using cross-validation. Based on the position-specific difference of amino acid compositions between S-nitrosylation sites and non-S-nitrosylation sites (Fig. 3), 21-mer (−10∼+10) is selected as the window length in the following evaluation and implementation. Herein, four types of feature including amino acid (AA), amino acid composition (AAC), accessible surface area (ASA), and 21 motifs – are evaluated. The feature of amino acids is encoded using a 20-dimensional vector and a positional weighted matrix, named “AA_20D” and “AA_PWM”, respectively. The features of accessible surface area (ASA) and motifs are encoded using the ASA values and 21-dimensional binary vector (Figure S1 in Supplementary Materials), respectively. According to the predictive accuracy given in Table 2, of the models trained with individual features, that trained with amino acid composition (AAC) slightly outperforms that trained with amino acid (AA_20D or AA_PWM), accessible surface area (ASA), or 21 motifs. However, the model trained with only ASA has the lowest predictive accuracy, which is probably caused by the low ASA value of cysteines.

**Table 2:** The cross-validation performance of the models trained with various features.

Training features	Sn	Sp	Pre	Acc	BAcc	MCC
Amino Acid (AA_20D)	0.556	0.574	0.199	0.572	0.566	0.097
Amino Acid (AA_PWM)	0.585	0.586	0.212	0.587	0.586	0.127
Amino Acid Composition (AAC)	0.579	0.605	0.218	0.602	0.593	0.137
Accessible Surface Area (ASA)	0.540	0.553	0.187	0.552	0.547	0.069
21 Motifs	0.556	0.563	0.195	0.562	0.560	0.088
**AA_PWM+AAC**	**0.640**	**0.681**	**0.277**	**0.675**	**0.661**	**0.245**
AA_PWM+ASA	0.561	0.583	0.204	0.580	0.573	0.108
AA_PWM+21 Motifs	0.561	0.572	0.199	0.570	0.567	0.098
AA_PWM+AAC+ASA	0.578	0.603	0.217	0.599	0.591	0.134
AA_PWM+AAC+21 Motifs	0.572	0.601	0.204	0.593	0.587	0.130
AA_PWM+AAC+ASA+21 Motifs	0.588	0.589	0.214	0.589	0.589	0.131

Abbreviation: AA_20D, amino acids coding with 20-dimensional vector; AA_PWM, positional weighted matrix of flanking amino acids; ASA, accessible surface area; Pre, precision; Sn, sensitivity; Sp, specificity; Acc, accuracy; BAcc, balanced accuracy; MCC, Matthews Correlation Coefficient.

Additionally, the predictive power of the model trained with the hybrid combination of AA, AAC, ASA, and 21 motifs is also evaluated. Amino acid (AA_PWM) is regarded as the basic feature for training a model with other features. As described previously, the feature of AAC yields the best accuracy of over 0.60. Therefore, as shown in Table 2, the model trained with a combination of AA_PWM and AAC perform best. The predictive sensitivity, specificity, accuracy, and Matthews Correlation Coefficient (MCC) of the best model are 0.640, 0.681, 0.675, and 0.245, respectively. However, the predictive power of the model trained with the combination of all features (AA_PWM, AAC, ASA and 21 motifs) is not better than that trained with AAC alone, presumably due to the features of ASA or 21 motifs performing not well for identifying S-nitrosylation sites.

**Figure 5:**
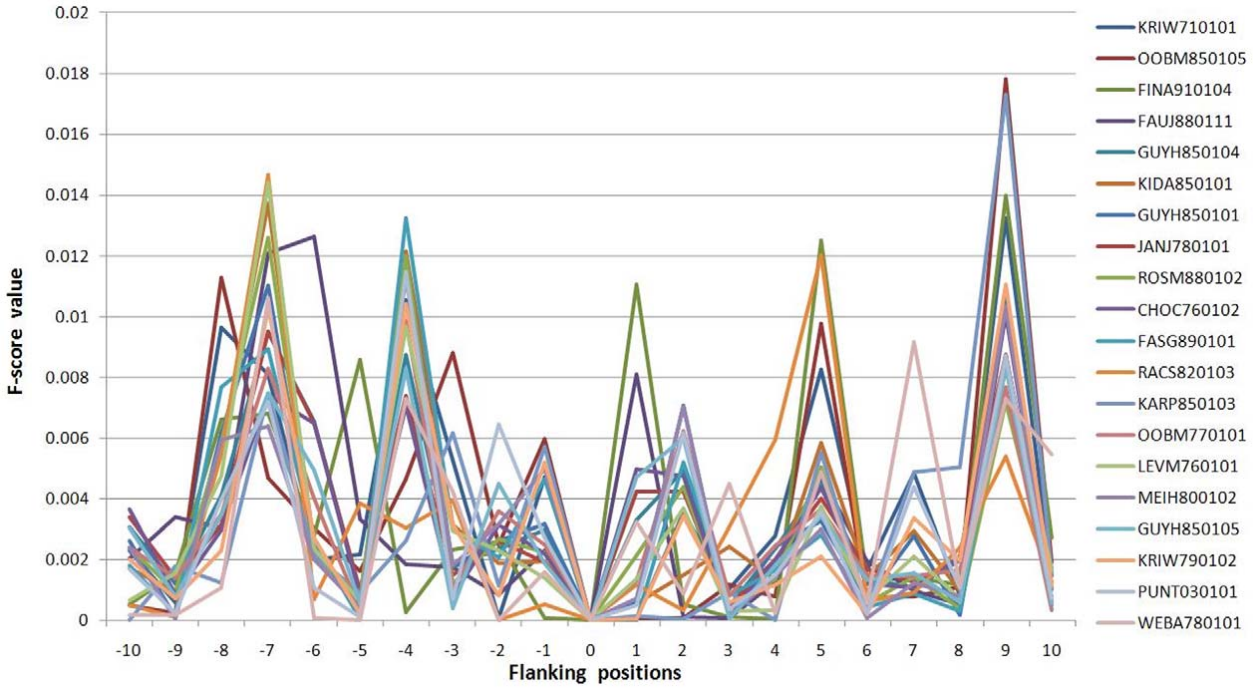
The top twenty physicochemical properties of S-nitrosylation sites ranked by the average value of F-score measurement in 21-mer window. KRIW710101, side chain interaction parameter [48]; OOBM850105, optimized side chain interaction parameter [49]; FINA910104, helix termination parameter at position j+1 [50]; FAUJ880111, positive charge [51]; GUYH850104, apparent partition energies calculated from Janin index [52]; KIDA850101, hydrophobicity-related index [53]; GUYH850101, partition energy [52]; JANJ780101, average accessible surface area [54]; ROSM880102, side chain hydropathy [55]; CHOC760102, residue accessible surface area in folded protein [56]; FASG890101, hydrophobicity index [57]; RACS820103, average relative fractional occurrence in AL(i) [58]; KARP850103, flexibility parameter for two rigid neighbors [59]; OOBM770101, average non-bonded energy per atom [60]; LEVM760101, hydrophobic parameter [61]; MEIH800102, average reduced distance for side chain [62]; GUYH850105, apparent partition energies calculated from Chothia index [52]; KRIW790102, fraction of site occupied by water [63]; PUNT030101, knowledge-based membrane-propensity scale from 1D_Helix in MPtopo databases [64]; WEBA780101, RF value in high salt chromatography [65].

To further analyze the physicochemical property of S-nitrosylation sites and adjacent amino acids, a total of 531 physicochemical properties, extracted from version 9.1 of AAindex [39], were individually explored [38]. Figure 5 shows the top twenty physicochemical properties ranked by the average value of F-score measurement in 21-mer window (−10∼+10). This investigation reveals that the twenty physicochemical properties contain high F-score values at positions −7, −4, +1, +2, +5, and +9, which have statistically significant difference between S-nitrosylation sites and non-S-nitrosylation sites. The predictive power of the twenty physicochemical properties was evaluated. According to the cross-validation performance of the models individually trained with each of the twenty physicochemical properties, the feature of positive charge perform better sensitivity, specificity, accuracy, balanced accuracy, and MCC than other physicochemical properties, which achieves an accuracy of about 0.60 (Table 3). The result was consisted with the position-specific difference of amino acid composition (Fig. 2), which contains positively charged amino acids neighboring to the S-nitrosylation sites. However, the model trained with the top ranking physicochemical property (side chain interaction parameter) is not performing as accurately as that trained with positive charge. Most of the twenty physicochemical properties reach an accuracy of about 0.55 (Table 3). Based on a concept of the forward feature selection, the ranked physicochemical properties can be sequentially added into the best model (AA_PWM+AAC) to evaluate whether the integration of physicochemical properties could improve the predictive performance. After evaluating the forward selection of top twenty physicochemical properties, the predictive power is not improved, when comparing to the model trained with AA_PWM and AAC (Figure S2 in Supplementary Materials).

**Table 3:** The cross-validation performance of the models trained individually with twenty physicochemical properties.

AAindex ID	Description	Sn	Sp	Pre	Acc	BAcc	MCC
KRIW710101	Side chain interaction parameter (Krigbaum-Rubin, 1971)	0.554	0.572	0.197	0.569	0.563	0.093
OOBM850105	Optimized side chain interaction parameter (Oobatake *et al.*, 1985)	0.547	0.560	0.191	0.558	0.554	0.079
FINA910104	Helix termination parameter at posision j+1 (Finkelstein *et al.*, 1991)	0.558	0.566	0.196	0.565	0.562	0.092
**FAUJ880111**	**Positive charge (Fauchere ** ***et al.*** **, 1988)**	**0.583**	**0.594**	**0.214**	**0.592**	**0.588**	**0.131**
GUYH850104	Apparent partition energies calculated from Janin index (Guy, 1985)	0.551	0.574	0.197	0.570	0.562	0.092
KIDA850101	Hydrophobicity-related index (Kidera *et al.*, 1985)	0.549	0.555	0.189	0.554	0.552	0.076
GUYH850101	Partition energy (Guy, 1985)	0.545	0.570	0.194	0.566	0.558	0.085
JANJ780101	Average accessible surface area (Janin *et al.*, 1978)	0.563	0.575	0.201	0.573	0.569	0.102
ROSM880102	Side chain hydropathy, corrected for solvation (Roseman, 1988)	0.540	0.545	0.183	0.544	0.542	0.062
CHOC760102	Residue accessible surface area in folded protein (Chothia, 1976)	0.565	0.582	0.204	0.579	0.574	0.109
FASG890101	Hydrophobicity index (Fasman, 1989)	0.535	0.560	0.187	0.556	0.547	0.069
RACS820103	Average relative fractional occurrence in AL(i) (Rackovsky-Scheraga, 1982)	0.511	0.533	0.172	0.530	0.522	0.033
KARP850103	Flexibility parameter for two rigid neighbors (Karplus-Schulz, 1985)	0.542	0.565	0.191	0.561	0.553	0.079
OOBM770101	Average non-bonded energy per atom (Oobatake-Ooi, 1977)	0.545	0.563	0.191	0.560	0.554	0.080
LEVM760101	Hydrophobic parameter (Levitt, 1976)	0.545	0.556	0.189	0.554	0.551	0.075
MEIH800102	Average reduced distance for side chain (Meirovitch *et al.*, 1980)	0.542	0.559	0.189	0.556	0.550	0.074
GUYH850105	Apparent partition energies calculated from Chothia index (Guy, 1985)	0.556	0.563	0.194	0.562	0.560	0.088
KRIW790102	Fraction of site occupied by water (Krigbaum-Komoriya, 1979)	0.531	0.554	0.184	0.550	0.542	0.062
PUNT030101	Knowledge-based membrane-propensity scale from 1D_Helix in MPtopo databases (Punta-Maritan, 2003)	0.543	0.566	0.192	0.562	0.555	0.081
WEBA780101	RF value in high salt chromatography (Weber-Lacey, 1978)	0.538	0.553	0.186	0.550	0.545	0.067

The physicochemical property that contains the highest accuracy is highlighted in bold. Abbreviation: Pre, precision; Sn, sensitivity; Sp, specificity; Acc, accuracy; BAcc, balanced accuracy; MCC, Matthews Correlation Coefficient.

### Exploring the potential S-nitrosylation motifs by MDD clustering

To improve the detection of the conserved motifs from large-scale S-nitrosylation data set,, we further apply the maximal dependence decomposition (MDD) to cluster all 586 identified S-nitrosylated peptide sequences into 11 subgroups, capturing the most significant dependencies of amino acid composition between positions. Table S3 (Supplementary Materials) shows the number of positive data in each subgroup and their average performances of five-fold cross-validations. According to the chi-square test of the dependence of five amino acid groups in flanking positions (Table S1), 10 out of all MDD-clustered subgroups have the conserved motifs of positively charged amino acids (K, R and H) at a specific position. In particular, the first and fourth subgroups have the negatively charged amino acids (D and E) accompanied by positively charged amino acids on conserved motifs at two specific positions, Consistent with the previous study [16], trans-nitrosylation by an intermediary nitrosothiol (RSNO) is catalytically assisted by neighboring H and D that act as base and acid, respectively. However, the eleventh subgroup, that contains 68 S-nitrosylation sites, does not have a conserved motif.

Furthermore, all of 11 MDD-clustered subgroups are evaluated for their predictive power for identifying S-nitrosylation sites, based on five-fold cross-validation. The number of negative data in each subgroup is determined according to the ratio of non-S-nitrosylation sites (2728 sequences) to S-nitrosylation sites (586 sequences) in training data. To avoid the skew sampling of negative data, ten sets of negative data, which are randomly selected from all non-S-nitrosylation sites, are constructed for each subgroup. Thus, in each subgroup, the predictive model is trained with the combined features of positional weighted matrix (AA_PWM) and amino acid composition (AAC), and the five-fold cross-validation is implemented ten rounds. The average value of cross-validation performance in each subgroup is displayed in Table S3 (Supplementary Materials). It indicates that most of the 11 subgroups could achieve an average accuracy of about 0.900. Especially, the first, second, fourth and tenth subgroups perform with an average accuracy of about 0.950, but the eleventh subgroup has the worst performance with an accuracy of 0.845. In conclusion, the average accuracy of all 11 subgroups is 0.902 which increases 0.227 predictive accuracy comparing to the model trained without MDD clustering. This analysis indicates that the S-nitrosylated sequences in a large-scale data set can be alternatively clustered by MDD method, which significantly enhanced the signal of amino acids motif and improved the performance of the predictive model.

### Evaluation of S-nitrosylation predictive models using independent test set

To evaluate effectiveness of the investigated features that achieves the best accuracy in cross-validation, an independent set is used to test the MDD-clustered models trained with positional weight matrix and amino acid compositions. The independent test set is composed of the experimentally verified S-nitrosylation data of GPS-SNO [26] from multiple species, which contains a total of 479 positive data and 2501 negative data in 327 S-nitrosylated proteins. As shown in Table 4, the MDD-clustered models could perform with an accuracy of 0.627 in all independent test set. Due to the various motifs in multiple predictive models, for all data of independent testing, the estimated sensitivity (0.805) is higher than specificity (0.593). Furthermore, the predictive performance is estimated for various types of species. For *E. coli*, the MDD-clustered models have the highest accuracy (0.730) with the balanced sensitivity (0.702) and specificity (0.736). Reasonably, the MDD-clustered models that were trained with S-nitrosoproteome data set from SNAP/L-cysteine-stimulated mouse endothelial cells have a high sensitivity (0.830) for 106 mouse S-nitrosylation sites. The proposed method also has high sensitivity (0.819) for 105 rat S-nitrosylation sites. Overall, the independent testing demonstrates that the MDD-clustered models have higher estimated sensitivity comparing to specificity.

**Table 4:** The predictive performance of MDD-clustered models using an independent test set (GPS-SNO).

Species	Number of proteins	Number of positive data	Number of negative data	TP	TN	FP	FN	Sn	Sp	Pre	Acc	BAcc	MCC
**All data**	327	479	2501	386	1485	1016	93	0.805	0.593	0.275	0.627	0.699	0.294
**Human**	117	211	1055	159	655	400	52	0.753	0.620	0.284	0.642	0.687	0.280
**Mouse**	84	106	568	88	378	190	18	0.830	0.665	0.316	0.691	0.747	0.366
**Rat**	70	105	597	86	385	212	19	0.819	0.644	0.288	0.670	0.731	0.334
**E. coli**	39	37	152	26	112	40	11	0.702	0.736	0.393	0.730	0.719	0.365
**ARATH**	5	5	36	5	5	31	0	1	0.138	0.138	0.243	0.569	0.138
**BOVIN**	2	5	34	5	4	30	0	1	0.117	0.142	0.230	0.558	0.129

Abbreviation: TP, true positive; TN, true negative; FP, false positive; FN, false negative; Pre, precision; Sn, sensitivity; Sp, specificity; Acc, accuracy; BAcc, balanced accuracy; MCC, Matthews Correlation Coefficient; ARATH, *Arabidopsis thaliana*; BOVIN, *Bos taurus*.

### Implementation of web-based tool for identifying S-nitrosylation sites

**Figure 6:**
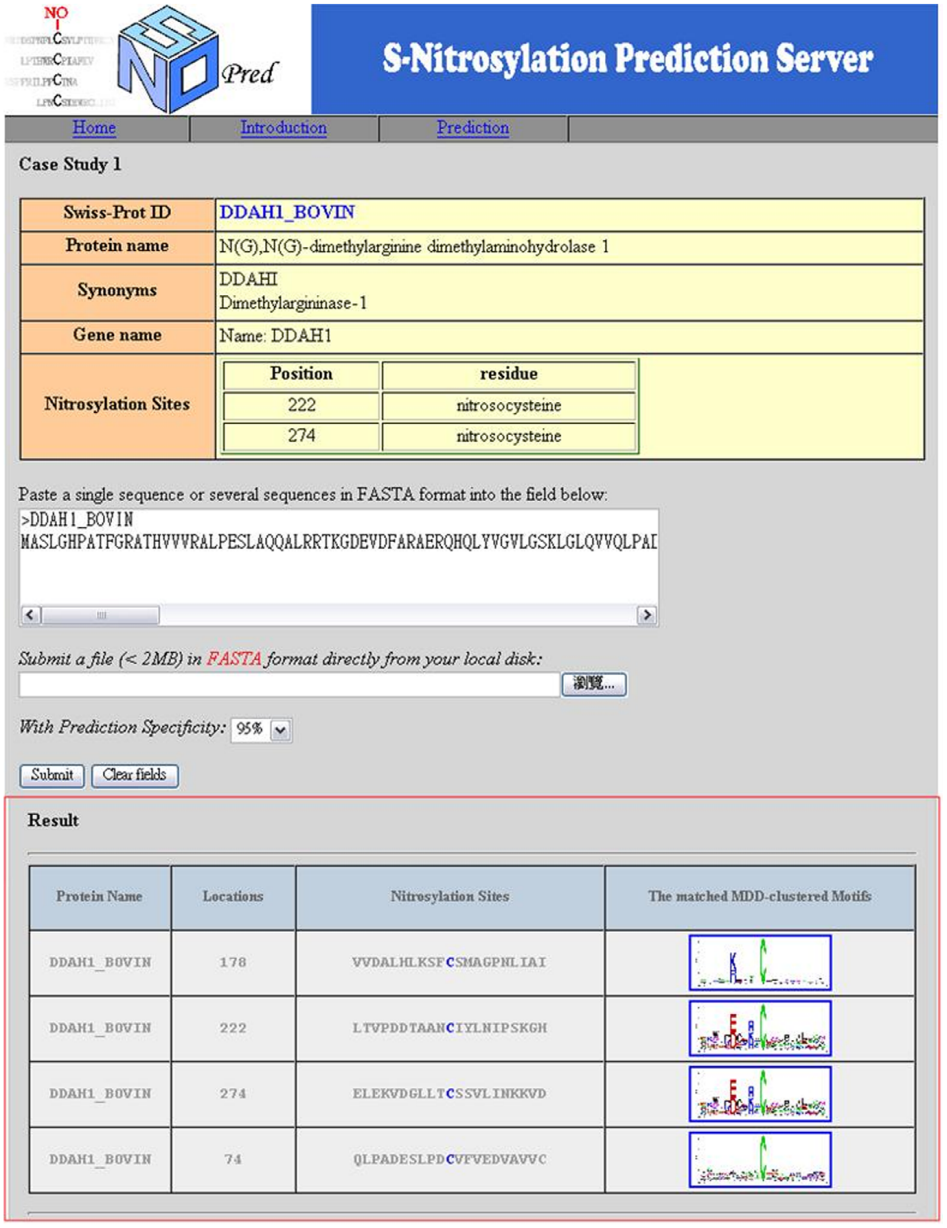
A case study of *Bos taurus* dimethylarginine dimethylaminohydrolase 1 (DDAH1) which contains two S-nitrosylation sites at positions 222 and 274.

With the time-consuming and laboratory-intensive experimental workflow, even though a protein can be S-nitrosylated, precise identification of the S-nitrosylation sites on the substrate is experimentally difficult. Therefore, an effective prediction tool should be developed to efficiently identify potential S-nitrosylation sites. Following evaluation by cross-validation and an independent test, the MDD-clustered models trained with positional weighted matrix of amino acids (AA_PWM) and amino acids composition (AAC) are utilized in the construction of web-based prediction system, SNOSite. After the users submit their uncharacterized protein sequences, SNOSite efficiently returns the predictions including S-nitrosylated position, the flanking amino acids, and the matched MDD-clustered motif. To demonstrate the performance of the tool, two experimentally-verified S-nitrosylated proteins which is not included in the training data set was studied. The first case study is performed on *Bos taurus* dimethylarginine dimethylaminohydrolase 1 (DDAH1) which contains two S-nitrosylation sites at positions 222 and 274 [45]. As presented in Fig. 6, SNOSite is able to correctly predict the experimentally verified S-nitrosylation site at positions 222 and 274. Additionally, two more cysteine residues are reported by SNOSite as novel S-nitrosylation sites. The matched MDD-clustered motifs are also provided for the future investigation of substrate site specificity. Next, the second case study was performed on human hemoglobin subunit beta (HBB) which contains one S-nitrosylation site at position 94 [46]. The experimentally verified S-nitrosylation site at position 94 was correctly predicted by SNOSite (Figure S3 is Supplementary Materials). In addition, one more cysteine residue is predicted as a novel S-nitrosylation site. SNOSite can be accessed via a web interface, and is freely available to all interested users at http://csb.cse.yzu.edu.tw/SNOSite/.

## Discussion

In this study, we reported a systematic informatics investigation on the S-nitrosylation substrate specificity from experimentally verified S-nitrosoproteome data [19]. The analysis of position-specific amino acids composition reveals that the most pronounced feature of S-nitrosylation sites is the abundance of positively charged amino acids at surrounding positions. This investigation also implicates that the distant amino acids in sequence (around position −7), which may be close to S-nitrosylation cysteines in three-dimensional structure, have notable difference between S-nitrosylation sites and non-S-nitrosylation sites. Additionally, the accessible surface area (ASA) and physicochemical properties are considered. Moreover, the S-nitrosylation sites have higher preference of solvent-accessible surface area, especially in the region of upstream sequences (−10∼−2). Based on the F-score measurement of 531 physicochemical properties in 21-mer window (−10∼+10), twenty physicochemical properties are revealed that contain statistically significant difference at positions −7, −4, +1, +2, +5, and +9 of S-nitrosylation sites. According to the five-fold cross-validation, the model trained with the combined features of positional weighted matrix and amino acids composition gets the highest accuracy.

Due to the abundance of experimental data, this study focuses on investigating the motifs of S-nitrosylation sites based on the amino acid sequences. However, it is difficult to explore the conserved motifs from large-scale S-nitrosoproteome data set. Thus, this work applies maximal dependence decomposition (MDD) to cluster all sequences of S-nitrosylation site into 11 subgroups, which have obvious motifs. According to the chi-square test of the dependence in flanking positions, most of the MDD-clustered subgroups have the conserved motifs of positively charged amino acids (K, R and H) at a specific position. Particularly, two subgroups have the conserved motifs of positively charged and negatively charged amino acids at two specific positions. Although the newly identified motifs could not be experimentally verified, what has to be noticed is MDD clustering can help the biologist investigating the potential substrate motifs of S-nitrosylation sites. More noteworthy is the MDD-clustered motifs can be applied to improve the predictive power of computationally identifying S-nitrosylation sites with various substrate specificities. According to the evaluation of five-fold cross-validation, the models trained with 11 MDD-clustered motifs increases predictive accuracy of 0.227 comparing to the model trained without MDD clustering. This analysis indicates that the S-nitrosylated sequences with a larger size can be alternatively clustered by MDD method in order to enhance the signal of amino acids motif and improve the performance of the predictive model. The models trained with MDD-clustered subgroups in overall perform better than that without MDD clustering. Consequently, the models with MDD clustering method are applied to implement a novel web-based tool, named SNOSite, for identifying cysteine S-nitrosylation. Correct prediction on two experimentally verified S-nitrosylated proteins demonstrated the effectiveness of SNOSite.

Furthermore, the experimental S-nitrosylation data of GPS-SNO [26] from multiple species is regarded as independent sets and is used to test the effectiveness of the models that achieve the best accuracy in cross-validation. Independent testing indicates that the model trained with MDD-clustered motifs could perform robustly for the test data from human, mouse, rat, and E. coli species. Although the proposed method can perform accurately and robustly according to independent tests, some issues should still be addressed in future work. Firstly, the structural preferences of S-nitrosylation sites should be investigated in greater detail - especially for the data whose flanking amino acids are not conserved. In addition to the solvent accessible surface area, secondary structure, the B-factor, intrinsic disordered region, protein linker region, and other factors should be examined at experimental S-nitrosylation sites which are located in the protein regions with PDB entries. Secondly, the biological function of S-nitrosylated proteins needs to be studied. The analysis of Gene Ontology [47] or the network of protein-protein interaction may provide a clue for inferring the function of S-nitrosylated proteins. Finally, the independent testing indicates that the predictive model could not perform well in part of test data that is not homologous to the training data. The acquisition of additional experimentally verified S-nitrosylation data is needed to re-calibrate more accurate MDD-clustered motifs. The proposed method can be improved by considering the motifs that are intrinsically included in the test data.

## Supporting Information

Figure S1 The coding scheme of 21 motifs for learning SVM classifier. A binary vector with 21 dimensions is used to denote what kind of motifs does a sequence has.
(TIF)

Figure S2 The predictive accuracy of the best model (AA_PWM+AAC) trained with forward selection of top twenty physicochemical properties.
(TIF)

Figure S3 A case study of human hemoglobin subunit beta (HBB) which contains one S-nitrosylation site at position 94.
(TIF)

Table S1 The grouping of amino acids used in MDD clustering.
(DOC)

Table S2 The 21 motifs of S-nitrosylation sites (586 sequences) identified by motif-x program with the parameters of motif occurrences and statistical significance are more than 10 and less than 0.01, respectively.
(DOC)

Table S3 The 11 MDD-clustered subgroups and their average performances of five-fold cross-validations.
(DOC)
