# Supplementary figures and images for: SNOSite: Exploiting Maximal Dependence Decomposition to Identify Cysteine S-Nitrosylation with Substrate Site Specificity
Source: PLoS One. 2011 Jul 15;6(7):e21849. doi: 10.1371/journal.pone.0021849 (PMC3137596; doi:10.1371/journal.pone.0021849)

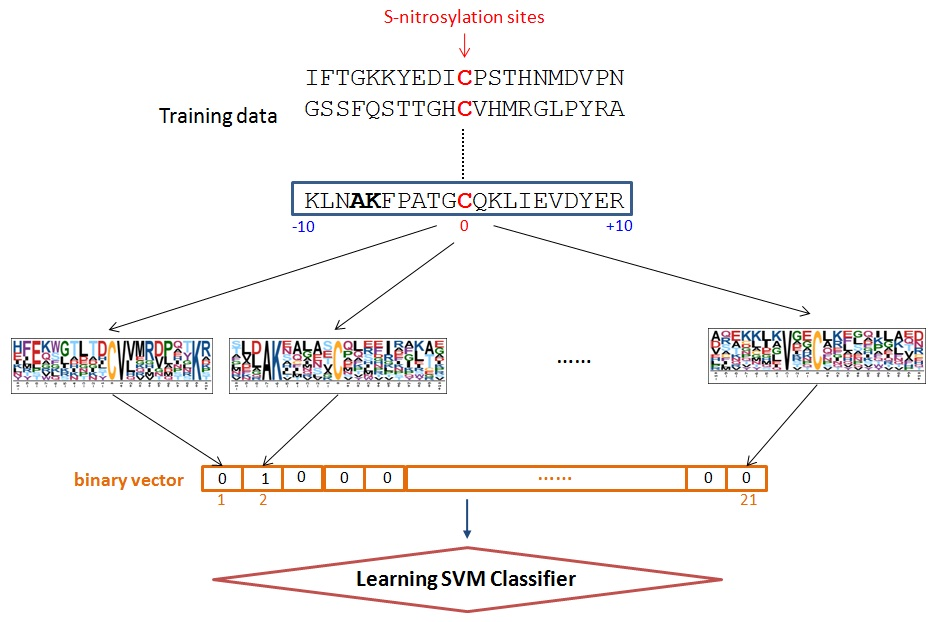

Supplement: Figure S1 — The coding scheme of 21 motifs for learning SVM classifier. A binary vector with 21 dimensions is used to denote what kind of motifs does a sequence has. (TIF) [file pone.0021849.s001.tif]

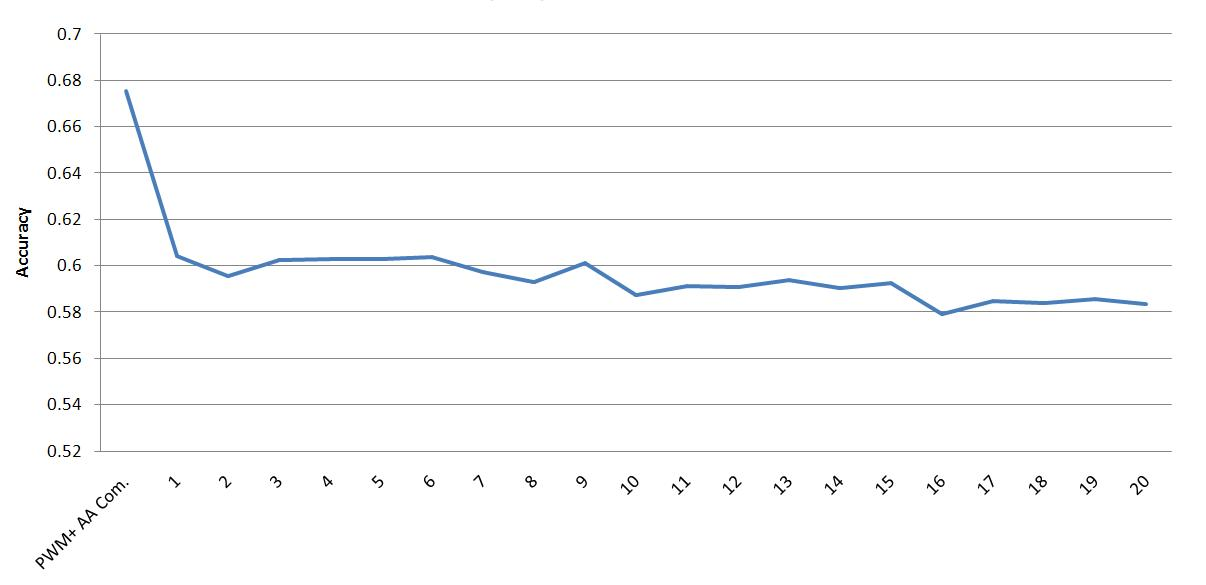

Supplement: Figure S2 — The predictive accuracy of the best model (AA_PWM+AAC) trained with forward selection of top twenty physicochemical properties. (TIF) [file pone.0021849.s002.tif]

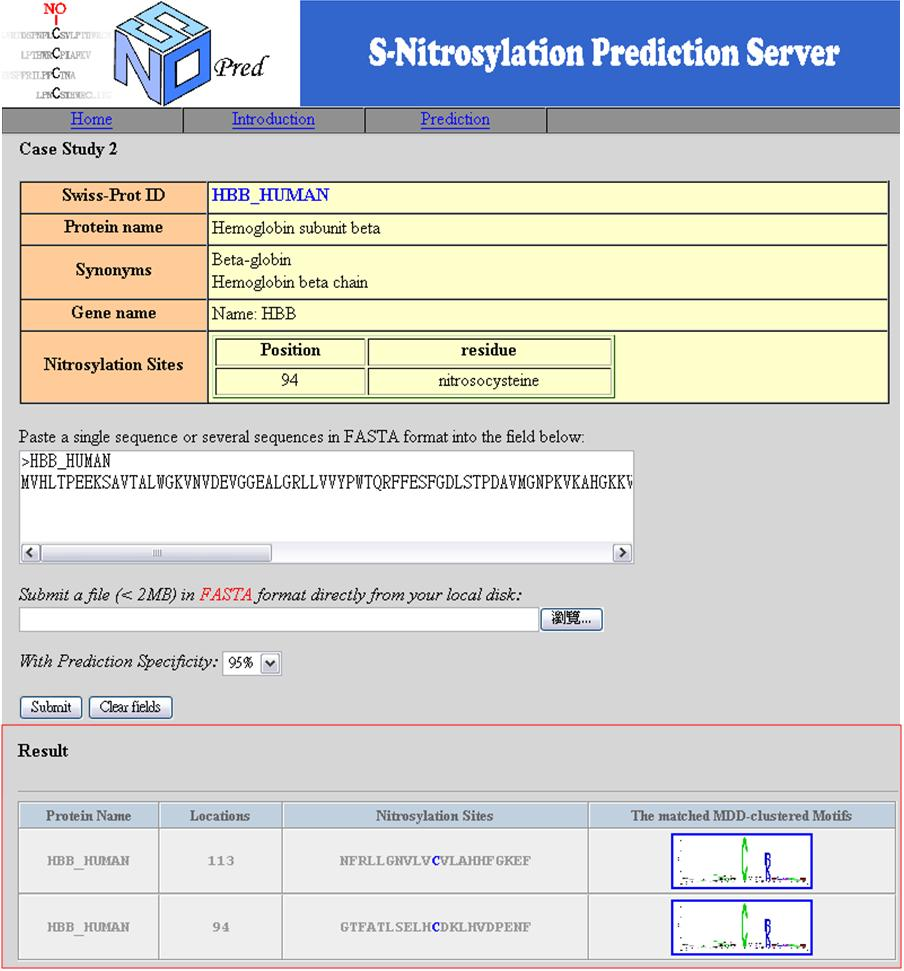

Supplement: Figure S3 — A case study of human hemoglobin subunit beta (HBB) which contains one S-nitrosylation site at position 94. (TIF) [file pone.0021849.s003.tif]
