# Supplementary material for: SNOSite: Exploiting Maximal Dependence Decomposition to Identify Cysteine S-Nitrosylation with Substrate Site Specificity
Source: PLoS One. 2011 Jul 15;6(7):e21849. doi: 10.1371/journal.pone.0021849 (PMC3137596; doi:10.1371/journal.pone.0021849)
Supplement: Table S1 — The grouping of amino acids used in MDD clustering. (DOC) [file pone.0021849.s004.doc]

**Table S1.** The grouping of amino acids used in MDD clustering.

| **Group name** | **Amino acids** |
| --- | --- |
| Aliphatic | glycine (G), alanine (A), valine (V), leucine (L), isoleucine (I), methionine (M) |
| Polar and uncharged | serine (S), threonine (T), cysteine (C), proline (P), asparagine (N), glutamine (Q) |
| Acid | aspartic acid (D), glutamic acid (E) |
| Basic | lysine (K), arginine (R), histidine (H) |
| Aromatic | phenylalanine (F), tyrosine (Y), tryptophan (W) |
