# Supplementary material for: SNOSite: Exploiting Maximal Dependence Decomposition to Identify Cysteine S-Nitrosylation with Substrate Site Specificity
Source: PLoS One. 2011 Jul 15;6(7):e21849. doi: 10.1371/journal.pone.0021849 (PMC3137596; doi:10.1371/journal.pone.0021849)
Supplement: Table S2 — The 21 motifs of S-nitrosylation sites (586 sequences) identified by motif-x program with the parameters of motif occurrences and statistical significance are more than 10 and less than 0.01, respectively. (DOC) [file pone.0021849.s005.doc]

**Table S2. The 21 motifs of S-nitrosylation sites (586 sequences) identified by motif-x program with the parameters of motif occurrences and statistical significance are more than 10 and less than 0.01, respectively.**

| **#** | **Motif Logo** | **Motif**  **Score** | **Foreground**  **Matches** | **Foreground**  **Size** | **Background**  **Matches** | **Background**  **Size** |
| --- | --- | --- | --- | --- | --- | --- |
| 1 | 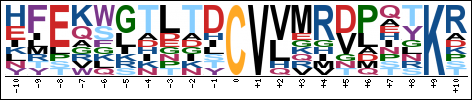 | 10.3 | 10 | 568 | 913 | 312726 |
| 2 | 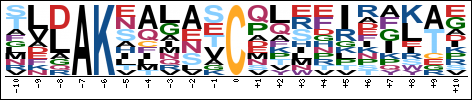 | 11.55 | 12 | 558 | 804 | 311813 |
| 3 | 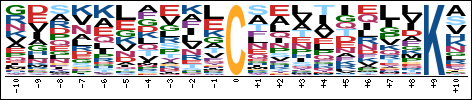 | 5.58 | 52 | 546 | 14856 | 311009 |
| 4 | 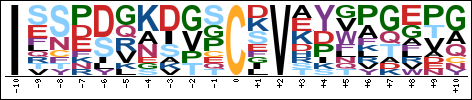 | 9.3 | 11 | 494 | 808 | 296153 |
| 5 | 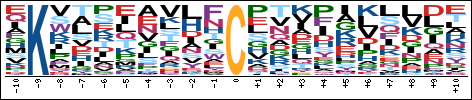 | 4.81 | 47 | 483 | 14832 | 295345 |
| 6 | 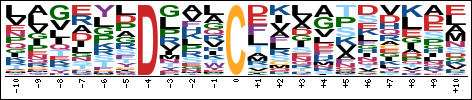 | 4.02 | 38 | 436 | 12599 | 280513 |
| 7 | 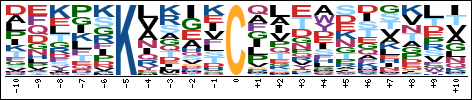 | 4.25 | 38 | 398 | 12869 | 267914 |
| 8 | 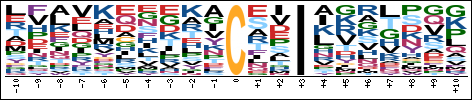 | 4.49 | 31 | 360 | 9807 | 255045 |
| 9 | 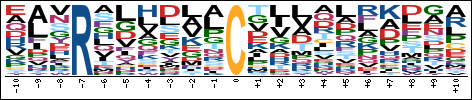 | 3.73 | 35 | 329 | 13556 | 245238 |
| 10 | 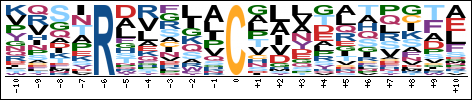 | 3.1 | 29 | 294 | 12036 | 231682 |
| 11 | 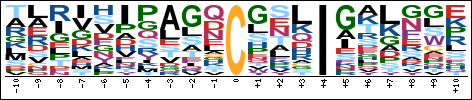 | 3.31 | 23 | 265 | 8796 | 219646 |
| 12 | 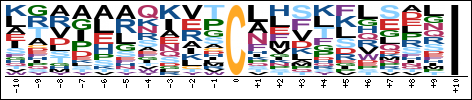 | 2.88 | 21 | 242 | 8770 | 210850 |
| 13 | 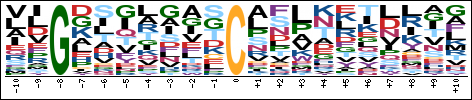 | 3.01 | 28 | 221 | 13570 | 202080 |
| 14 | 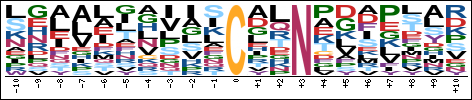 | 2.99 | 17 | 193 | 7044 | 188510 |
| 15 | 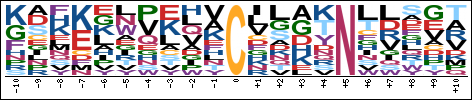 | 2.58 | 14 | 176 | 6094 | 181466 |
| 16 | 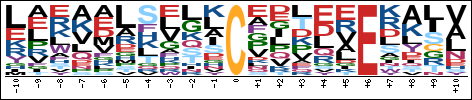 | 2.79 | 19 | 162 | 9676 | 175372 |
| 17 | 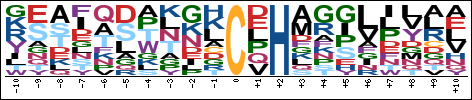 | 2.89 | 12 | 143 | 4955 | 165696 |
| 18 | 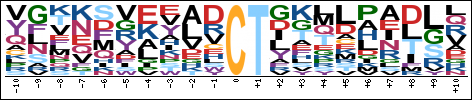 | 2.64 | 15 | 131 | 7997 | 160741 |
| 19 | 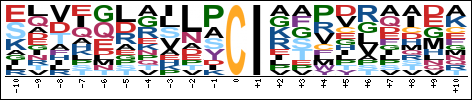 | 2.85 | 13 | 116 | 6528 | 152744 |
| 20 | 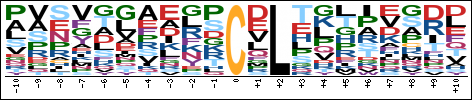 | 2.56 | 20 | 103 | 14578 | 146216 |
| 21 | 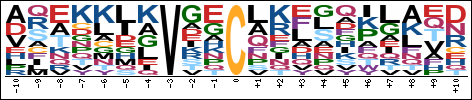 | 2.25 | 12 | 83 | 8276 | 131638 |
