# Supplementary material for: SNOSite: Exploiting Maximal Dependence Decomposition to Identify Cysteine S-Nitrosylation with Substrate Site Specificity
Source: PLoS One. 2011 Jul 15;6(7):e21849. doi: 10.1371/journal.pone.0021849 (PMC3137596; doi:10.1371/journal.pone.0021849)
Supplement: Table S3 — The 11 MDD-clustered subgroups and their average performances of five-fold cross-validations. (DOC) [file pone.0021849.s006.doc]

**Table S3**. The 11 MDD-clustered subgroups and their average performances of five-fold cross-validations. Abbreviation: Pre, precision; Sn, sensitivity; Sp, specificity; Acc, accuracy; BAcc, balanced accuracy; MCC, Matthews Correlation Coefficient.

| **Group** | **Number of data** | | **Sequence logo** | **Sn** | **Sp** | **Pre** | **Acc** | **BAcc** | **MCC** |
| --- | --- | --- | --- | --- | --- | --- | --- | --- | --- |
| 1 | Positive | 25 | 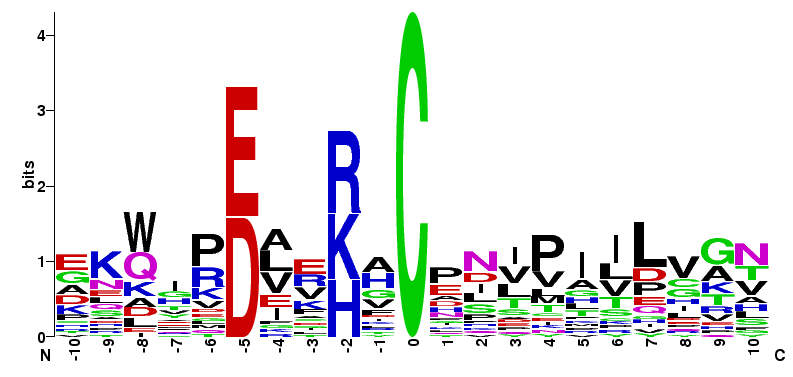 | 0.840 | 0.956 | 0.807 | 0.935 | 0.898 | 0.784 |
|  | Negative | 115 |  |  |  |  |  |  |  |
| 2 | Positive | 42 | 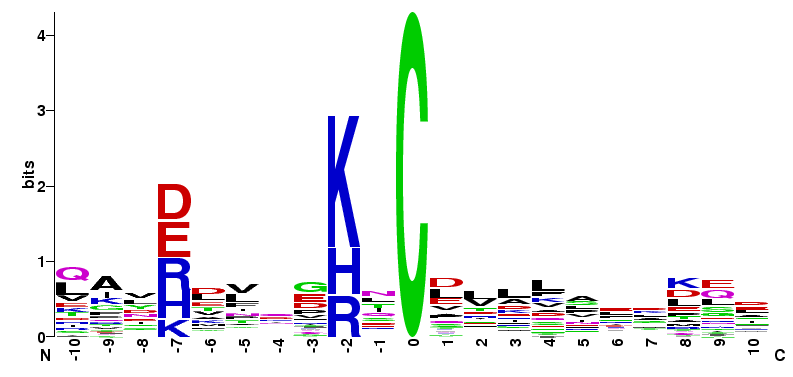 | 0.952 | 0.932 | 0.754 | 0.936 | 0.942 | 0.811 |
|  | Negative | 193 |  |  |  |  |  |  |  |
| 3 | Positive | 61 | 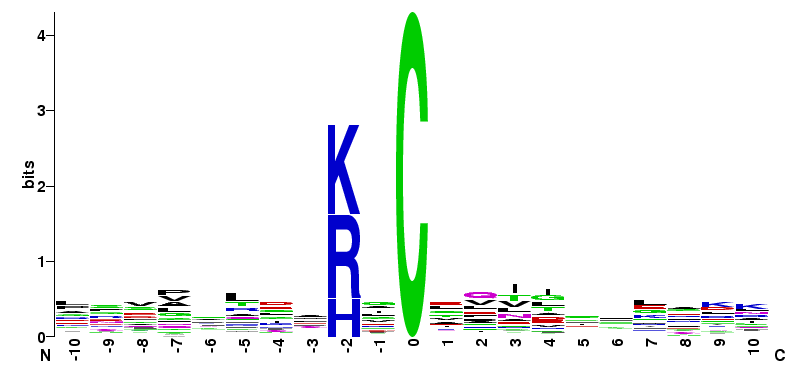 | 0.918 | 0.896 | 0.658 | 0.900 | 0.907 | 0.721 |
|  | Negative | 280 |  |  |  |  |  |  |  |
| 4 | Positive | 20 | 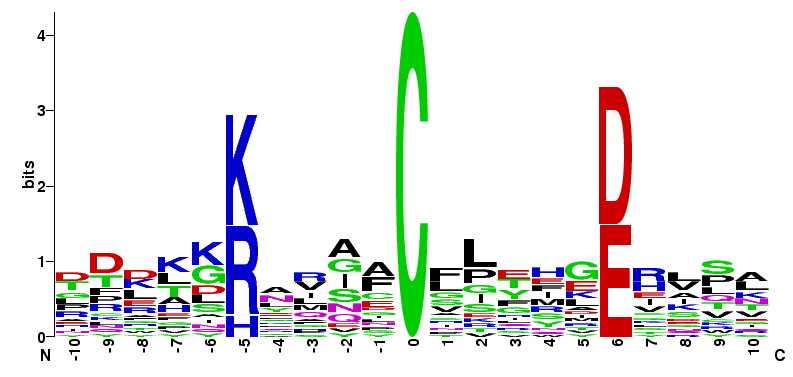 | 0.950 | 0.956 | 0.826 | 0.955 | 0.953 | 0.859 |
|  | Negative | 92 |  |  |  |  |  |  |  |
| 5 | Positive | 94 | 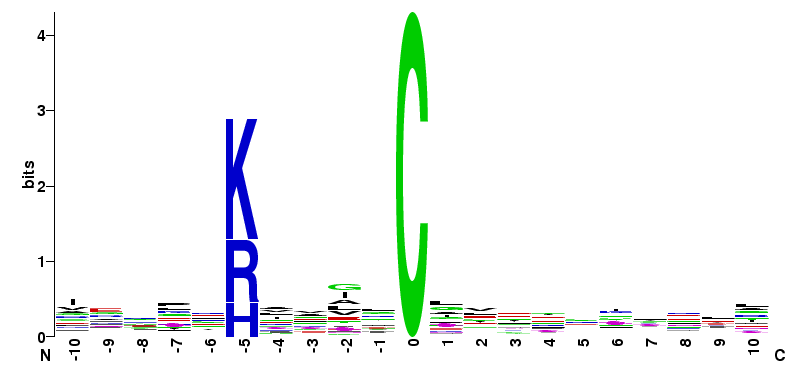 | 0.851 | 0.884 | 0.615 | 0.878 | 0.867 | 0.653 |
|  | Negative | 432 |  |  |  |  |  |  |  |
| 6 | Positive | 87 | 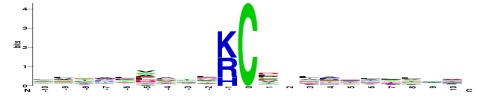 | 0.839 | 0.897 | 0.640 | 0.887 | 0.868 | 0.666 |
|  | Negative | 400 |  |  |  |  |  |  |  |
| 7 | Positive | 72 | 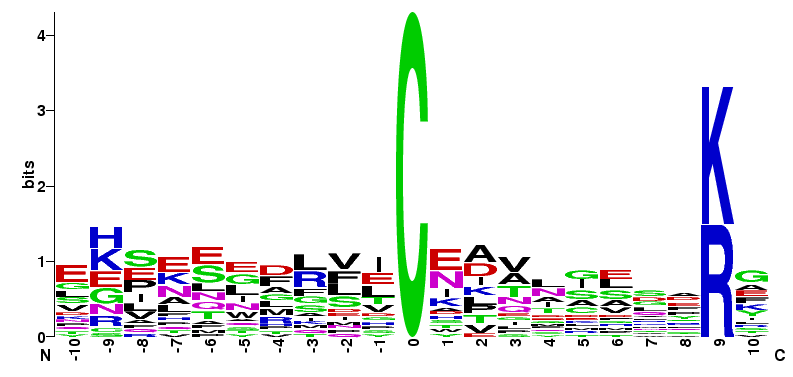 | 0.861 | 0.879 | 0.607 | 0.875 | 0.870 | 0.652 |
|  | Negative | 331 |  |  |  |  |  |  |  |
| 8 | Positive | 47 | 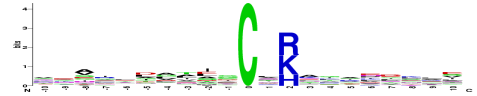 | 0.872 | 0.861 | 0.577 | 0.863 | 0.866 | 0.632 |
|  | Negative | 216 |  |  |  |  |  |  |  |
| 9 | Positive | 34 | 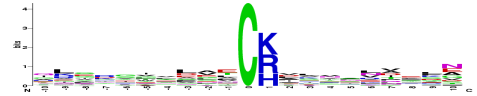 | 0.911 | 0.910 | 0.688 | 0.910 | 0.911 | 0.741 |
|  | Negative | 156 |  |  |  |  |  |  |  |
| 10 | Positive | 26 | 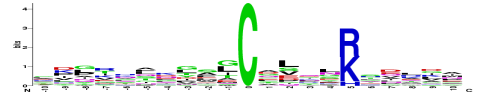 | 0.923 | 0.949 | 0.800 | 0.944 | 0.936 | 0.826 |
|  | Negative | 119 |  |  |  |  |  |  |  |
| 11 | Positive | 68 | 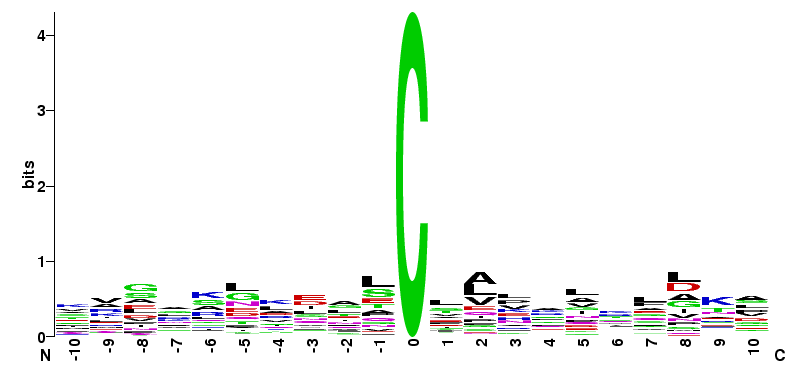 | 0.838 | 0.846 | 0.542 | 0.845 | 0.842 | 0.586 |
|  | Negative | 313 |  |  |  |  |  |  |  |
| **Average** | | |  | **0.887** | **0.906** | **0.683** | **0.902** | **0.896** | **0.721** |
